# An optimal normalization method for high sparse compositional microbiome data

**DOI:** 10.1371/journal.pcbi.1012338

**Published:** 2024-08-05

**Authors:** Michael B. Sohn, Cynthia Monaco, Steven R. Gill

**Affiliations:** 1 Biostatistics and Computational Biology, University of Rochester Medical Center, Rochester, New York, United States of America; 2 Medicine, University of Rochester Medical Center, Rochester, New York, United States of America; 3 Microbiology and Immunology, University of Rochester Medical Center, Rochester, New York, United States of America; Genome Institute of Singapore, SINGAPORE

## Abstract

In many omics data, including microbiome sequencing data, we are only able to measure relative information. Various computational or statistical methods have been proposed to extract absolute (or biologically relevant) information from this relative information; however, these methods are under rather strong assumptions that may not be suitable for multigroup (more than two groups) and/or longitudinal outcome data. In this article, we first introduce the minimal assumption required to extract absolute from relative information. This assumption is less stringent than those imposed in existing methods, thus being applicable to multigroup and/or longitudinal outcome data. We then propose the first normalization method that works under this minimal assumption. The optimality and validity of the proposed method and its beneficial effects on downstream analysis are demonstrated in extensive simulation studies, where existing methods fail to produce consistent performance under the minimal assumption. We also demonstrate its application to real microbiome datasets to determine biologically relevant microbes to a specific disease/condition.

## Author summary

Microbiome sequencing samples are not directly comparable to each other, as the sequencing depth (i.e., the total count of sequencing reads in a sample) varies substantially. Thus, making samples comparable is an essential part in microbiome data analysis. The most common approach is to transform read counts into proportions. However, this transformation introduces unwanted effects, known as compositional effects (i.e., a change in abundance of a microbe leads to changes in abundance of all other microbes), as proportions must sum to one. Therefore, direct comparison of individual microbes between groups can produce many false positives. Rarefaction also provokes these compositional effects, as the sequencing depth for all samples is fixed at a constant. In this article, we propose a novel method that can remove the compositional effects under a less stringent assumption, thus being able to identify biologically relevant information from relative information in multigroup and/or longitudinal outcome data.

## Introduction

In almost every investigated disease, different microbial communities have been found between healthy and diseased groups. [1–4] These findings have led basic and clinical researchers to investigate the potential of the human microbiome as clinical treatment or intervention and develop microbiome-based therapies. For example, *Clostridioides difficile* diarrhea occurs due to its overgrowth after disruption of the normal gut microbiota, and fecal microbiota transplant (FMT) is highly efficacious and now widely used for recurrent or recalcitrant diarrhea due to *C. difficile* infection. [5] This success has prompted several clinical trials examining the impact of FMT in treatment of inflammatory bowel disease (IBD) and prevention of IBD flares. [6–9] However, FMT requires donations of feces from carefully screened volunteers. Despite careful screening, adverse outcomes, including death, have occurred from the transmission of pathogenic enteric bacteria and antibiotic resistant bacteria during FMT for recurrent *C. difficile* infection, [10–12] prompting an FDA warning in 2020. [13] This raises concerns for similar adverse impact with this therapy in IBD patients, many of whom may be on strong immunosuppressive regimens to control disease and therefore at higher risk of infectious complications. Better knowledge of discriminant microbes (or taxa) associated with a specific disease would allow more specific, improved therapeutic options to target only taxa involved in the disease and decrease risk of transmission of pathogenic bacteria, as well as allow more mechanistic studies focusing on specific taxa. Therefore, identifying biologically, differentially abundant (DA) taxa, some of which can be causal, is essential in translating human microbiome studies to clinical practice. However, this has been hindered in part by the compositional nature of microbiome data.

**Fig 1:**
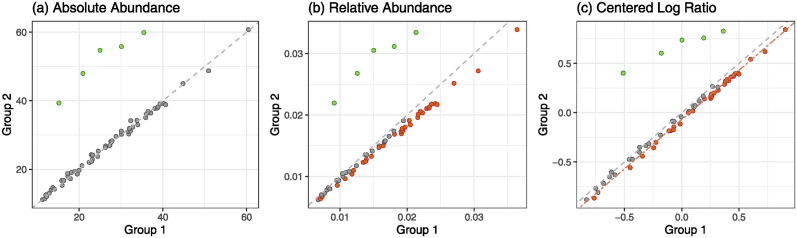
An illustration of a compositional effect on differential abundance analysis. With mean counts of 60 taxa, among which 5 taxa are truly, differentially abundant between two groups. Fig (a) shows their true mean counts between two groups. Only 5 taxa (green) are truly, differentially abundant. Fig (b) shows a compositional effect, i.e., a consequence of transforming counts into proportions, where many non-DA taxa (red) in addition to the 5 true DA taxa are detected as DA taxa. Fig (c) shows mitigated but still unresolved the compositional effect after the CLR transformation. The Wilcoxon rank-sum test followed by the Benjamini-Hochberg correction was used to determine DA taxa at FDR ≤ 0.05.

Microbiome sequencing data is usually organized into a count matrix of taxa, where rows represent samples and columns represent taxa. The total count of taxa (or sequencing depth) in a sample varies considerably, and this substantial variation typically does not reflect the biological variation. To remove this non-biological variation, thus making samples comparable, counts are typically transformed into proportions before any downstream analysis. Since proportions in a sample must sum to one (known as a *unit-sum constraint*), this transformation can create a compositional effect. Fig 1 illustrates this compositional effect on differential abundance analysis (i.e., determining discriminant taxa in abundance between groups). Only five taxa (green-color points) are truly DA taxa, as shown in Fig 1(a); however, many non-DA taxa (red-color points) are found to be DA taxa due to the compositional effect, as shown in Fig 1(b). Note that rarefaction has the same issue as it randomly subsamples taxa to a pre-specified sequencing depth. To resolve this compositional effect, various log-ratio transformations have been adopted, such as an additive log-ratio (ALR) or a centered log-ratio (CLR) transformation. [14] The CLR transformation, which uses the geometric mean of proportions as a reference in log-ratio, mitigates but does not resolve the compositional effect, as shown in Fig 1(c). LinDA [15] tries to reduce these false positives by correcting the bias caused by the CLR transformation, i.e., the perpendicular distance between the gray and red dotted lines in Fig 1(c). To this end, they assume the majority of taxa are non-DA taxa, which is a common assumption used in many other methods, including RAIDA [16] and RDB [17]. This assumption may be reasonable for two-group comparison but may be strong for multi-group comparison.

An alternative strategy for making samples comparable is to determine a size factor for each sample, which reflects the sequencing depth of a sample, and rescale counts using the size factor. Since it is not constrained to a specific value, this strategy does not suffer from the unit-sum constraint. The robustness of the estimated size factor, however, relies on the assumption(s) used in the methods under this strategy. For instance, trimmed mean of M values (TMM) [18], which was developed for RNA-Seq data and adapted to many omics data, including microbiome data, assumes the upper and lower trimmed taxa in M value (i.e., log fold change) are irrelevant to the condition (e.g., healthy or diseased) under study. Cumulative sum scaling (CSS) [19] assumes the taxa whose abundance levels are lower than a data-driven percentile of the abundance level are irrelevant to the condition under study. A recent approach implemented in DACOMP [20] uses a similar strategy of TMM and CSS. All these methods perform satisfactorily when their assumptions are satisfied. However, their assumptions may be strong and difficult, if not impossible, to be justified, especially for multigroup and/or longitudinal outcome microbiome data.

A seemingly promising approach to this challenge in removing the compositional effects is to use cell-based (e.g., flow cytometry) or DNA-based (e.g., quantitative polymerase chain reaction or qPCR) methods. [21–24] This approach first determines the count of all taxa or a specific taxon in each sample using flow cytometry or qPCR and then uses it as a reference to normalize samples. However, this approach has its own limitations besides substantial additional costs. Flow cytometry, for instance, requires considerable expertise for reproducible results, and qPCR assumes 100% lysis in the first step of DNA extraction, [22] thus being prone to substantial variation in the estimated size factor. In other words, a gain from the cell- or DNA-based method may be marginal compared to other aforementioned approaches.

In this paper, we first introduce the minimal assumption required to remove the compositional effects, without an additional complex and costly procedure. This minimal assumption is less stringent than those imposed in existing methods (e.g., the majority of microbes are not differentially abundant), so it can be applicable to multigroup comparison and/or longitudinal data. We then propose an optimal method for normalization (OPTIMEM) that can be used to remove the compositional effects under the minimal assumption. We demonstrate its accuracy and validity in selecting a subset of non-DA taxa under some regulatory conditions (e.g., signals are stronger than noise levels) theoretically and empirically. We demonstrate its beneficial effects on downstream analysis, particularly in differential abundance analysis, in extensive simulation studies. Using the proposed method, we also reanalyze several real data: an inflammatory bowel disease (IBD) dataset [25], a human immunodeficiency virus (HIV) dataset [26], and an upper respiratory tract (URT) dataset [27].

## Materials and methods

*Rationale of the proposed method*: If we can determine a subset of non-DA taxa, we use their sum as a reference (or denominator) in ratio to remove the compositional effects.

### Definitions and notations

We here summarize some definitions of operators proposed by Aitchison [14] and notations used in this manuscript. Let C denote the constraining operator that transforms $w\in R_{+}^{p}$ into $w/\left(j_{p}^{⊤}w\right)\in S^{\left(p-1\right)}$, where ***j***_*p*_ is the length *p* vector of ones, $R_{+}^{p}$ is the positive orthant of *p*-dimensional real space, and $S^{p-1}$ is the (*p* − 1)-dimensional simplex, i.e., the constraining operator transforms counts into proportions. Let S be a selecting matrix of order *q* × *p*, with *q* elements equal to 1, one in each row and at most one in each column, and the remaining elements 0, where *q* < *p*. In other words, S selects *q* parts from a *p*-part composition. We denote by A an amalgamating matrix of order *q* × *p*, with *p* elements equal to 1, one in each column and at least one in each row, and the remaining elements 0. The amalgamating matrix transforms a *p*-part composition into a *q*-part composition by amalgamating parts. We denote an *n*_*g*_ × *p* matrix of the *g*-th group taxonomic profile by *M*_*g*_ and an *n* × *p* matrix of taxonomic profile consisting of *G* groups by $M={\left(M_{1}^{⊤},\ldots ,M_{G}^{⊤}\right)}^{⊤}$, where *n*_*g*_ is the number of observations in the *g*-th group and $n=\sum_{g=1}^{G}n_{g}$.

### Minimal assumption

It is impossible to obtain absolute (or biologically relevant) information from relative abundance without any assumptions as C(w)=C(a×w), where *a* is any positive constant, e.g., C(1,4,5)=(0.1,0.4,0.5)=C(2,8,10). The most common assumption imposed by existing methods is that the majority of taxa are not differentially abundant across groups, which is a rather strong assumption, especially for multigroup and/or longitudinal outcomes. In this manuscript, we establish the minimal assumption required for any computational or statistical method that attempts to extract absolute from relative information (or compositional data). Let ***μ***_*g*_ = (*μ*_*g*1_, …, *μ*_*gp*_)^⊤^ be a vector of the true abundance levels of taxa in ecosystem *g*, i.e., $\mu_{g}=Ew_{g}$, where $w_{g}\in R_{+}^{p}$. Let $N=\{j\;|\;\mu_{1j}=\cdots =\mu_{Gj};\;j=1,\ldots ,p\}$ be the set of the true non-DA taxa across G ecosystems, and $\left\{D_{1},\ldots ,D_{S}\right\}$ is the set of all possible subsets of subcompositionally equivalent DA taxa, i.e., $C\left(\mu_{1j\in D_{s}}\right)=\cdots =C\left(\mu_{Gj\in D_{s}}\right)$ and *s* = 1, …, *S*. The minimal assumption required to extract biologically relevant information from compositional data is given by:

$$\underset{s}{\text{max}}{\left\|D_{s}\right\|}_{0}<{\left\|N\right\|}_{0},$$

where ‖⋅‖_0_ counts the number of elements in a set. This assumption basically requires that the number of non-DA taxa is greater than the number of subcompositionally equivalent DA taxa, which is satisfied with a high probability as *p* increases. To illustrate this assumption, let’s consider a simple example, where we have five taxa in two groups with their true counts, ***μ***_1_ = (5, 4, 3, 2, 2) and ***μ***_2_ = (10, 8, 6, 2, 2), i.e., the last two taxa are non-DA taxa. Since we can only measure the relative abundance of these taxa, what we would observe is ***μ***_1_ = (0.31, 0.25, 0.19, 0.13, 0.13) and ***μ***_2_ = (0.36, 0.29, 0.21, 0.07, 0.07). In these proportions, the maximum number of subcompositionally equivalent DA taxa is 3 (the first three taxa) since C(0.31,0.25,0.19)=C(0.36,0.29,0.21), thus ${\text{max}}_{s}{\left\|D_{s}\right\|}_{0}=3>{\left\|N\right\|}_{0}=2$, violating the assumption. However, it is satisfied if ***μ***_1_ = (5, 4, 3, 2, 2) and ***μ***_2_ = (10, 6, 8, 2, 2) as ${\text{max}}_{s}{\left\|D_{s}\right\|}_{0}=1<{\left\|N\right\|}_{0}=2$, whereas the majority non-DA taxa assumption is violated. Note that this assumption implies that there exist some non-DA taxa across groups in a dataset to be analyzed, which is necessary to avoid comparing non-comparable samples, e.g., samples in different sites.

### Approach

To find a subset of non-DA taxa under the minimal assumption, we sequentially remove a random subset of taxa that likely contains DA taxa until only a subset of non-DA taxa is left with a high probability. Specifically, let *k* be an index for the *k*-th removal sequence and *η* be a prespecified proportion of taxa removed at each removal sequence. We denote by *M*^(*k*)^ a matrix containing (1 − *η*)^*k*^ × 100% of taxa at removal sequence *k*, with *M*^(0)^ = *M*. Let *b* = 1, …, *B* be an index for a set of randomly selected taxa, and denote a matrix containing the *b*-th set of the taxa at the *k*-th removal sequence after removing *η* × 100% of taxa from *M*^(*k*−1)^ by

$$\begin{array}{c} M^{\left(k,b\right)}=M^{\left(k-1\right)}S^{(b)⊤}, \end{array}$$ (1)

where $S^{\left(b\right)}$ denotes the *b*-th ⌊(1 − *η*)^*k*^*p*⌋ × ⌊(1 − *η*)^*k*−1^*p*⌋ random selecting matrix, where ⌊⋅⌋ is the floor function. When *η* ≤ *p*^−1^, $S^{\left(b\right)}$ selects all taxa in *M*^(*k*−1,*b*)^ except the *b*-th one, and *b* = 1, …, *p*^(*k*−1)^, where *p*^(*k*−1)^ is the number of taxa in *M*^(*k*−1,*b*)^. In this case, *M*^(1,1)^ is, for instance, a taxonomic profile with the first taxon removed.

#### Naïve approach

To determine a subset of non-DA, we can determine indices *k* and *b* satisfying:

$$\left(\hat{k},\hat{b}\right)=\underset{k,b}{\text{argmin}}\;W^{\left(k,b\right)}\;\;\text{subject}\;\text{to}\;\;c_{l}\leq W^{\left(k,b\right)}\leq c_{u},$$

where *c*_*l*_ and *c*_*u*_ are prespecified cutoffs, such as a lower and an upper bound of a null empirical distribution of *W*^(1,*b*)^ obtained by randomly permuting group membership, and

$$W^{\left(k,b\right)}=\sum_{i=1}^{G}\sum_{j=i+1}^{G}{\left[C({\bar{m}}_{i}^{\left(k,b\right)})-C({\bar{m}}_{j}^{\left(k,b\right)})\right]}^{2},$$

where ${\bar{m}}_{g}^{\left(k,b\right)}$ is a vector of the mean abundance of taxa in $M_{g}^{\left(k,b\right)}$. *W*^(*k*,*b*)^ basically measures the squared mean differences in composition between all pairwise groups at sequential removal step (*k*, *b*). Thus, in noiseless settings, *W*^(*k*,*b*)^ becomes zero when all DA taxa are removed since $C({\bar{m}}_{1}^{\left(k,b\right)})=C({\bar{m}}_{2}^{\left(k,b\right)})=\cdots =C({\bar{m}}_{G}^{\left(k,b\right)})$. However, the performance of this naïve approach is not satisfactory due to the high-dimensionality and high-sparsity (i.e., high proportion of zeros) of microbiome data, which add high uncertainty in the estimate of mean composition.

#### Innovative approach

To resolve the issues in the naïve approach, we propose an innovative sequential removal and random amalgamation procedure. Specifically, we repeatedly construct an *n* × 2 matrix by amalgamating randomly selected taxa from *M*^(*k*, *b*)^. We denote this matrix by

$$\begin{array}{c} M^{\left(k,b,r\right)}=M^{\left(k,b\right)}A^{(r)⊤}, \end{array}$$ (2)

where $A^{\left(r\right)}$ is a random 2 × ⌊(1 − *η*)^*k*^*p*⌋ amalgamating matrix if *η* is greater than *p*^−1^ or a random 2 × (*p* − *k*) amalgamating matrix otherwise, where *r* = 1, …, *R*. We denote the sample mean of log-ratios for group *g* at sequential removal step (*k*, *b*) and random amalgamation step *r* by $x_{g}^{\left(k,b,r\right)}\equiv j_{g}^{⊤}\;\text{log}\left(M_{g}^{\left(k,b,r\right)}\right)\iota /n_{g}$, where ***j***_*g*_ is a length *n*_*g*_ vector of ones and $\iota ={\left(1,-1\right)}^{⊤}$. Note that this random amalgamation procedure removes the high-dimensionality and mitigates the high-sparsity substantially. In noiseless settings, $x_{1}^{\left(k,b,r\right)}=\cdots =x_{G}^{\left(k,b,r\right)}$ for any *r* if *M*^(*k*,*b*)^ contains no DA taxa. In other words, we can stop the sequential removal when $x_{1}^{\left(k,b,r\right)}=\cdots =x_{G}^{\left(k,b,r\right)}$ for any *r*, as the taxa in *M*^(*k*,*b*)^ are all non-DA taxa. For general noise settings, we propose the following criterion to determine indices *k* and *b*:

$$\begin{array}{c} \left(\hat{k},\hat{b}\right)=\underset{k,b}{\text{argmin}}\;Q^{\left(k,b\right)}\;\;\text{subject}\;\text{to}\;\;\delta_{l}\leq Q^{\left(k,b\right)}\leq \delta_{u}, \end{array}$$ (3)

where $Q^{\left(k,b\right)}=j_{g}^{⊤}D^{\left(k,b\right)}j_{g}$ is the mean sum of squared differences in log-ratio (MSS) between all pairwise groups at sequential removal step (*k*, *b*); *δ*_*l*_ and *δ*_*u*_ are prespecified cutoffs; and

$$\begin{array}{c} D^{\left(k,b\right)}=[\text{diag}\left(H^{\left(k,b\right)}\right)j_{G}^{⊤}+j_{G}\text{diag}{\left(H^{\left(k,b\right)}\right)}^{⊤}-2H^{\left(k,b\right)}]/2R, \end{array}$$ (4)

where

$$\begin{array}{c} H^{\left(k,b\right)}=[\begin{array}{cccc} \left\langle x_{1}^{\left(k,b\right)},x_{1}^{\left(k,b\right)}\right\rangle & \left\langle x_{1}^{\left(k,b\right)},x_{2}^{\left(k,b\right)}\right\rangle & \cdots & \left\langle x_{1}^{\left(k,b\right)},x_{G}^{\left(k,b\right)}\right\rangle \\ \left\langle x_{2}^{\left(k,b\right)},x_{1}^{\left(k,b\right)}\right\rangle & \left\langle x_{2}^{\left(k,b\right)},x_{2}^{\left(k,b\right)}\right\rangle & \cdots & \left\langle x_{2}^{\left(k,b\right)},x_{G}^{\left(k,b\right)}\right\rangle \\ \vdots & \vdots & \ddots & \vdots \\ \left\langle x_{G}^{\left(k,b\right)},x_{1}^{\left(k,b\right)}\right\rangle & \left\langle x_{G}^{\left(k,b\right)},x_{2}^{\left(k,b\right)}\right\rangle & \cdots & \left\langle x_{G}^{\left(k,b\right)},x_{G}^{\left(k,b\right)}\right\rangle \end{array}], \end{array}$$ (5)

where $x_{g}^{\left(k,b\right)}={\left(x_{g}^{\left(k,b,1\right)},\ldots ,x_{g}^{\left(k,b,R\right)}\right)}^{⊤}$. When *η* ≤ *p*^−1^, $Q^{\left(k,\hat{b}\right)}$ would decrease as *k* increases if the signal-to-noise ratio (SNR) is greater than one, where $\hat{b}$ indicates the optimal index for *b* at removal sequence *k*. However, as *k* increases, the number of the remaining taxa decreases, thus being prone to getting unstable estimates due to high sparsity, which may cause an increase in $Q^{\left(k,\hat{b}\right)}$. To find an optimal index for *k*, we propose a GAP statistic-type approach [28]. Specifically, we randomly reassign group membership and estimate the range of *Q*^(1,*b*)^ to construct a null distribution of MSS, denoted by *MSS*_0_. We stop the sequential removal if MSS is smaller than the empirical mean or the *α*-th percentile of *MSS*_0_. The corresponding $M^{\left(\hat{k},\hat{b}\right)}$ will contain only a subset of non-DA taxa with a high probability under some regularity conditions.

*Remark 1: If there is no DA-taxa, all MSS will be within the confidence interval of MSS*_0_ *at a pre-specified δ significance level with probability* 1 − *δ. Note that MSS at a large k can be larger than the upper limit of MSS*_0_ *in finite samples due to high-sparsity*.

*Remark 2: If all taxa are DA, all MSS will be larger than the upper limit of MSS*_0_.

**Fig 2:**
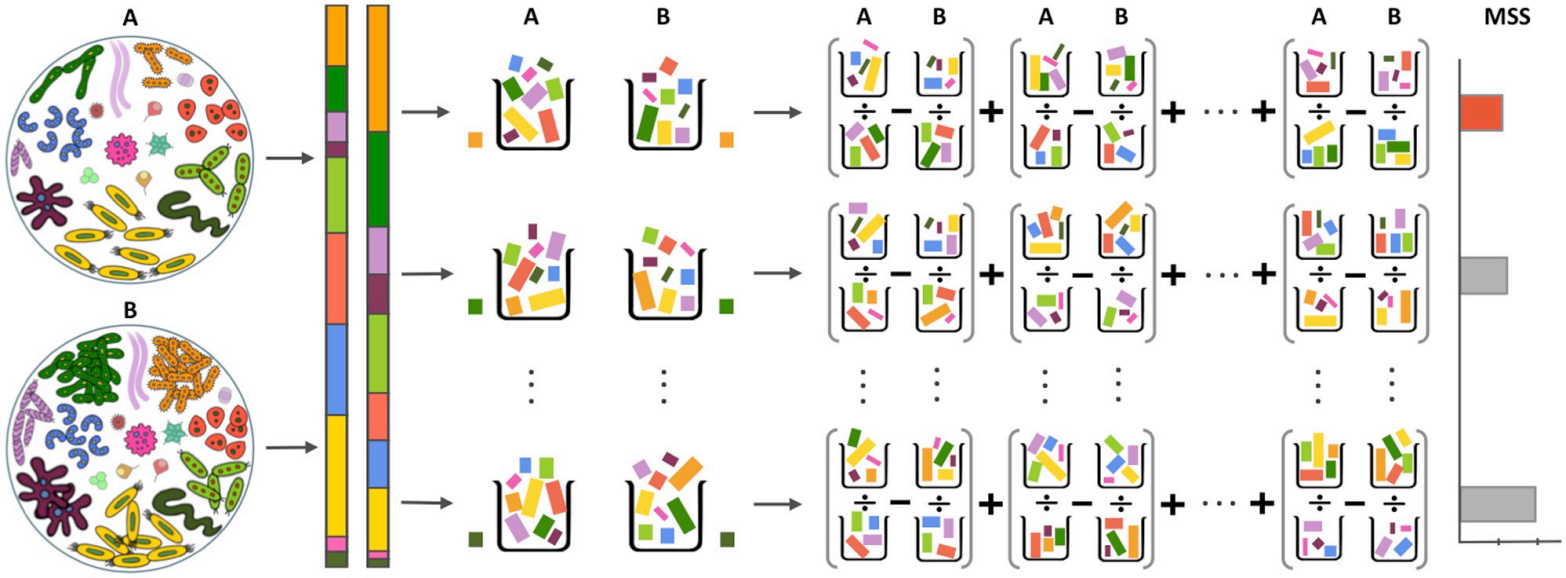
Graphical description of OPTIMEM for two groups. The rescaled relative abundance of taxa in ecosystems A and B at the start of the *k*-th sequential removal and random amalgamation step is shown in the stacked bar plot, where the first four from the top are DA taxa. Each row in the third column depicts the *b*-th sequential removal step, and each difference in ratios in the fourth column depicts the *r*-th random amalgamation step. The mean sum squared log-ratios (MSS) is the smallest when *b* = 1, i.e., when the first taxon (orange color) was removed. Thus, the first taxon will be excluded in the (*k* + 1)-th step. This procedure will stop when there is no significant difference in MSS between two consecutive sequential removal and random amalgamation steps.

A graphical description of this method for two groups in the *k*-th removal step is shown in Fig 2, where ten taxa are left at the start of the *k*-th removal step. Among these taxa, only the first four taxa from the top of the stacked bar plots are DA taxa. Note that the constraining operator was applied to the ten taxa (i.e., their sum becomes 1) to emphasize the compositional effect, but it is unnecessary in implementation as rescaling has no effects in ratio. OPTIMEM sequentially removes one taxon at a time and computes the corresponding MSS. It then removes the taxon with the smallest MSS. In the example, the first taxon is removed at the end of the *k*-th iteration as it has the smallest MSS. This sequential removal and random amalgamation procedure is repeated until the smallest MSS satisfies a stopping criterion, as described in Algorithm 1.

**Algorithm 1** Sequential Removal and Random Amalgamation

1: Construct a distribution of MSS_0_ and initialize MSS with a big number

2: Set *k* = 1, *η*, *B*, *R*, and a stopping criterion (e.g., the 5-th percentile of MSS_0_)

3: **repeat**

4:  **for** *b* = 1 to *B* **do**

5:   Randomly select *η* × 100% taxa from *M*^(*k*−1)^ using (1)

6:   **for** *r* = 1 to *R* **do**

7:    Randomly amalgamate to build an *n* × 2 matrix using (2)

8:    **for** *g* = 1 to *G* **do**

9:     Compute log-ratios $x_{g}^{\left(k,b,r\right)}$ for each group

10:    **end for**

11:   **end for**

12:   Compute $Q^{\left(k,b\right)}=j_{g}^{⊤}D^{\left(k,b\right)}j_{g}$ using (4) and (5)

13:   **if** *Q*^(*k*, *b*)^ < MSS **then**

14:    Update MSS ← *Q*^(*k*, *b*)^ and $\left(\hat{k},\hat{b}\right)\leftarrow \left(k,b\right)$

15:   **end if**

16:  **end for**

17:  Update *k* ← *k* + 1

18: **until** MSS ≤ a stopping criterion | the number of remaining taxa < 10% taxa

**Fig 3:**
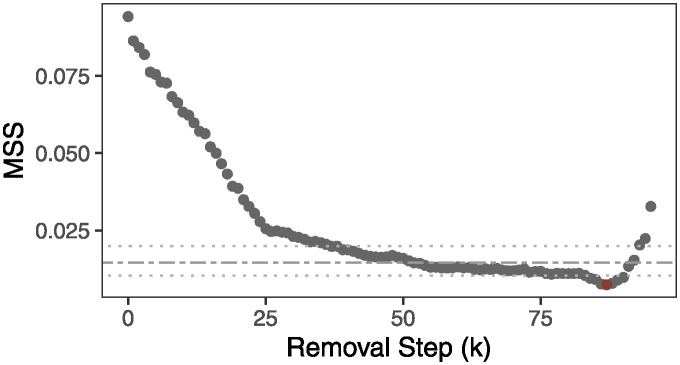
A Gap statistic-type approach to an optimal *k*. The two-dash line indicates the mean, and two dotted lines indicate the upper and lower limits of MSS_0_ at *α* = 0.05.

Fig 3 shows a result of the GAP statistic-type approach. Theoretically, any MSS below an upper bound of MSS_0_ can be used for the stopping criterion for *k*. However, MSS below a lower bound of MSS_0_, if reached, would provide a better interpretation of the remaining taxa, as we can quantify the probability of not having DA taxa in the remaining taxa more precisely. An asymptotic property of this procedure is provided in Theorem 1 (Asymptotic Property), and a proof of this theorem is given in S1 Text.

**Theorem 1** (Asymptotic Property) *Let η* ≤ *p*^−1^ *such that each removal sequence* (*k*, *b*) *removes just one taxon. Assuming the mean of log-ratios* $x_{g}^{\left(k,b,r\right)}$ *is well-approximated by a normal distribution with finite mean and finite variance*, $P(Q^{\left(\hat{k},b\right)}>Q^{\left(\hat{k},\hat{b}\right)})\to 1$ *with sufficiently large n*_*g*_ *and R*.

Theorem 1 states that when reaching a point where only one DA taxon is left in the remaining taxa, OPTIMEM can determine a subset of non-DA taxa with high probability, given sufficiently large *n*_*g*_ and *R* such that SNR > 1. Note that *R* is a prespecified value and can be set as large as necessary unlike *n*_*g*_, which is fixed for a given dataset. Theorem 1 does not state the asymptotic behavior when there are multiple DA taxa in the remaining taxa because it is possible that removing a non-DA taxon can have a smaller MSS than removing any DA taxa. However, this possibility becomes smaller as *k* increases and MSS decreases, as demonstrated empirically in simulation studies.

**Fig 4:**
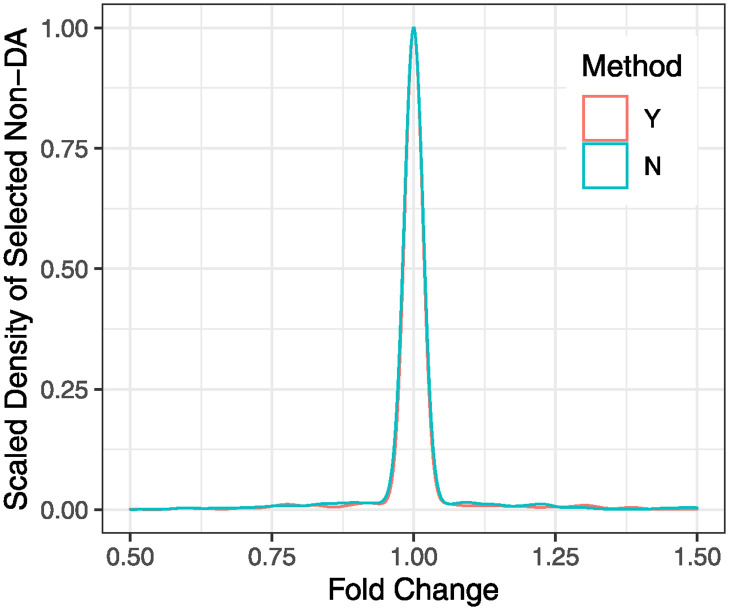
Scaled densities of selected non-DA taxa for the two approaches (using or not using group membership) with respect to the fold change in abundance of a taxon between two groups, based on NB models. Y indicates using group membership and N indicates not using group membership.

A subset of non-DA taxa can be determined without using group membership by adding an iterative random sample selection procedure to select the partition of samples with the largest $Q^{\left(1,\hat{b}\right)}$. However, this additional iteration step will increase the computational cost substantially. For more than two group outcomes, it may not be computationally feasible. Alternatively, an unsupervised clustering analysis, such as k-medoids clustering, which can approximate this additional iteration step, can be applied. In this manuscript, using k-medoids clustering with a prespecified cluster size, we empirically show the asymptotic equivalence of the two approaches (i.e., using and not using group membership). See Fig 4 for their similarities in non-DA taxa selection.

## Results

### Simulation studies

We first demonstrate the performance of OPTIMEM in selecting a subset of non-DA taxa, which will be used as a reference in ratio to remove compositional effects. We then show its beneficial effects on downstream analysis, particularly in differential abundance analysis. We simulated taxonomic profiles in various settings using three models: negative binomial (NB) models, which generate the abundance of each taxon independently; logistic normal (LN) models, which generate the abundance of taxa jointly, i.e., each taxon is correlated to each other; a permuted real microbiome dataset, which generates null models. We also simulated taxonomic profiles in two different settings: the balance case and the unbalance case. The former is where the sums of DA taxa across groups are similar, thus having negligible or weak compositional effects. The latter is where the sums of DA taxa across groups are substantially different, thus having strong compositional effects. Details of model parameters and simulation settings are given in S2 Text. For OPTIMEM, we used *R* = 1000 and *η* < *p*^−1^ in all simulation studies. The following methods were considered in performance comparison in differential abundance analysis: ANCOMBC [29], LinDA [15], RAIDA [16], RDB [17], and DACOMP [20], which were developed specifically to address the compositional effect; metagenomeSeq2 [19], ANCOM [30], edgeR [31], and the Wilcoxon rank-sum (WR) or Kruskal-Wallis (KW) test after rarefying samples [32–34], which are commonly used methods representing log-ratio, count, or proportion based approaches. In performance comparison, like rarefaction, OPTIMEM was followed by the WR or KW test. For multiple testing correction, the Benjamini-Hochberg (BH) procedure was used for all methods. [35] As some methods only produce which taxa are DA or non-DA taxa, we used the true positive rate (TPR) and the false discovery rate (FDR) as performance comparison measures.

#### Merits of OPTIMEM in identification of a subset of Non-DA Taxa

In this simulation study, we assessed the performance of OPTIMEM in selecting a subset of non-DA taxa with and without using group membership. We randomly selected a number of DA taxa from 5 to 50 out of 100 taxa and randomly generated their mean counts ***μ***^(*g*)^ for two groups *g* = 1, 2. We then simulated a taxonomic profile using NB models with these mean counts. We first ran OPTIMEM with their group membership and then reran it with the cluster membership determined by k-medoids clustering with a pre-specified number of clusters (i.e., 2 clusters). In each run, a sample size of 50 was used for each group. Fig 4 shows the scaled densities of selected non-DA taxa using the two approaches with respect to the fold change in abundance of each taxon between two groups, based on 100 repetitions. The two approaches show almost identical results, indicating that a subset of non-DA taxa can be determined with or without using group membership. As shown in Fig 4, if the fold changes of taxa are greater than 1.1 or less than 0.9, the probability of selecting these taxa as non-DA taxa is close to zero. Note that a fold change between 0.9 and 1.1 is smaller than noise levels and thus cannot be distinguished from no fold change (i.e., 1.0) with the sample size used in this study. Similar results were observed when taxonomic profiles were simulated using LN models, as shown in S1 Fig.

For the permuted real microbiome dataset (i.e., the IBD dataset) in which any association between the outcome (i.e., diagnoses) and each taxon was removed, OPTIMEM identified all taxa as non-DA taxa in all 100 repetitions.

#### Effects of OPTIMEM on differential abundance analysis for binary outcomes

*Favorable Settings for Existing Methods*. We first evaluated how well the proposed approach (i.e., OPTIMEM + a differential abundance analysis method) performed in settings where the performance of some existing methods was supposed to be optimal as their assumptions were satisfied. Specifically, we randomly generated a number of DA taxa from 5 to 25 out of 100 taxa and simulated a taxonomic profile using an NB or LN model in the balance or unbalance case. We repeated 100 times for each model. For OPTIMEM and rarefaction, the WR test was used for a differential abundance analysis method. When taxonomic profiles were simulated using NB models in the balance case, all methods performed comparably in both TPR and FDR, as shown in S2 Fig. However, the methods that do not account for the compositionality (e.g., edgeR, metagenomeSeq, rarefaction) failed to control FDR when taxonomic profiles were simulated using LN models, even in the balance case, as shown in S3 and S4 Figs. These methods also failed to control FDR when taxonomic profiles were simulated using NB models in the unbalance case, as shown in S5 Fig. Even in all these favorable settings to existing methods, the proposed approach performed consistently, although not significantly, better than existing methods.

*Antagonistic Settings for Existing Methods*. In this simulation study, we assessed the performance of the proposed approach in settings where the performance of some existing methods might severely deteriorate. To construct these antagonistic settings, we artificially created three simulation scenarios. The first scenario consisted of subsets of DA taxa that were subcompositionally equivalent between two groups in the balance case. The second scenario was the same as the first one, except it was in the unbalance case. The last scenario was a setting where the majority non-DA taxa assumption is violated in the unbalance case. The performance of the methods on the three scenarios is summarized in Table 1. Note that the first two scenarios do not violate the assumptions of any existing methods. However, the performance of some methods was substantially affected when the subcompositionally equivalent sets of DA taxa were present. When the majority non-DA taxa assumption was violated (Scenario 3), none of the existing methods controlled FDR. For some methods, their FDRs were even greater than their TPRs. The proposed approach, on the other hand, provided a very robust performance in all three scenarios. It was the only method that controlled FDR at a given level (FDR ≤ 0.05) while achieving good TPRs in all three scenarios.

**Table 1:** Performance comparison under antagonistic settings for existing methods. OPTIMEM and rarefaction indicate OPTIMEM and rarefaction followed by the WR test, respectively. The BH correction was used for multiple testing at FDR ≤ 0.05.

Method	Scenario 1		Scenario 2		Scenario 3	
	TPR	FDR	TPR	FDR	TPR	FDR
OPTIMEM	0.71	0.04	0.95	0.05	0.98	0.03
RDB	0.30	0.01	0.61	0.03	0.00	1.00
ANCOM	0.18	0.05	0.07	0.25	0.03	0.52
ANCOMBC	0.52	0.03	0.57	0.17	0.23	0.71
LinDA	0.64	0.07	0.92	0.06	0.16	0.82
DACOMP	0.32	0.14	0.42	0.40	0.28	0.67
edgeR	0.78	0.06	0.73	0.52	0.88	0.40
metagenomeSeq2	0.55	0.08	0.51	0.45	0.38	0.56
RAIDA	0.38	0.13	0.49	0.34	0.18	0.80
rarefaction	0.58	0.03	0.61	0.42	0.92	0.17

Scenario 1: the presence of subcompositionally equivalent sets of DA taxa in the balance case. Scenario 2: the presence of subcompositionally equivalent sets of DA taxa in the unbalance case. Scenario 3: a violation of the majority non-DA taxa assumption in the unbalance case.

#### Effects of OPTIMEM on differential abundance analysis for tertiary outcomes

**Fig 5:**
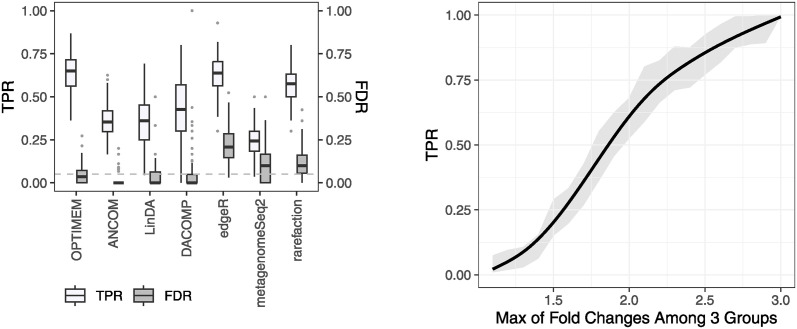
TPR vs FDR, and the performance of the proposed approach at different fold changes. The sample size was 50 per group, and the number of taxa was 100. The results were based on 100 repetitions. OPTIMEM and rarefaction indicate OPTIMEM + KW and rarefaction + KW, respectively. The left figure shows the comparison results when LN models were used to simulate taxonomic profiles with the majority non-DA constraint. The right figure shows the performance of the proposed method given the maximum fold change of a taxon among three groups without the majority non-DA constraint.

In this simulation study, we assessed the performance of the proposed approach in three-group outcomes. We first simulated taxonomic profiles using NB or LN models with mixing the balance and unbalance cases. The number of DA-taxa across three groups was randomly selected from {10, 11, …, 45} out of 100 taxa. The sample size was 50 per group. For OPTIMEM and rarefaction, the KW test was used for a differential abundance analysis method. Notice that this setting does not violate any assumption of existing methods. Fig 5 shows the comparison results for taxonomic profiles simulated using LN models. The proposed approach had the highest TPR while controlling FDR. Rarefaction and edgeR had good TPRs but did not control FDR. One noticeable result was the poorer performance of LinDA, compared to its relatively good performance on the binary outcome. Similar results were observed when taxonomic profiles were simulated using NB models, as shown in S6 Fig. RDB, ANCOMBC, and RAIDA were not included in comparison because they can be applied only to binary outcomes.

We then simulated taxonomic profiles without imposing the majority non-DA taxa assumption. As existing methods showed very poor performance in the binary group study when the majority non-DA taxa assumption was violated, we only measured the performance of the proposed approach at different fold changes, as shown in Fig 5, where fold changes were inverted if they were less than 1. In 100 repetitions, the range of non-DA taxa was (30, 78), and the mean (sd) number was 53 (11) across three groups. The mean (sd) proportion of fold changes that are between 0.5 and 2.0 for DA taxa across three groups was 0.33 (0.08). When a fold change of a DA taxon between any two groups was greater than 2 (or smaller than 0.5), the proposed approach achieved a substantial power while controlling FDR. The mean of TPRs was 0.72, and the mean of FDR was 0.03. These results are very similar to the binary outcome results, demonstrating the consistent performance of the proposed approach in multigroup outcomes under the minimal assumption.

Lastly, we permuted the IBD dataset to construct null models and measured the false positive rate (FPR) of each method, except for edgeR which constantly issued errors. All methods controlled FPR at *α* = 0.05 but metagenomeSeq whose FPR was 0.21.

### Real data analysis

#### Inflammatory bowel disease

IBD is a chronic inflammatory disease of the gastrointestinal tract impacting over 3 million adults in the US, and it is comprised of ulcerative colitis (UC) and Crohn’s disease (CD). [6] Symptoms can range from bloody diarrhea with abdominal pain to severe disease, including the development of strictures, fistula formation, intestinal perforation, and death. The IBD dataset contains shotgun metagenomic sequencing of stool samples from 53 UC, 68 CD, and 34 non-inflammatory disease (non-IBD) subjects. Franzosa *et al*. [25] applied a multivariable linear model to a log-transformed abundance of each of the 195 species that were present in at least five samples at 0.1% relative abundance, with age as a continuous covariate and four medications (antibiotics, immunosuppressants, mesalamine, and steroids) as binary covariates. They identified 50 DA species in one or more diagnoses (UC, CD, non-IBD).

**Fig 6:**
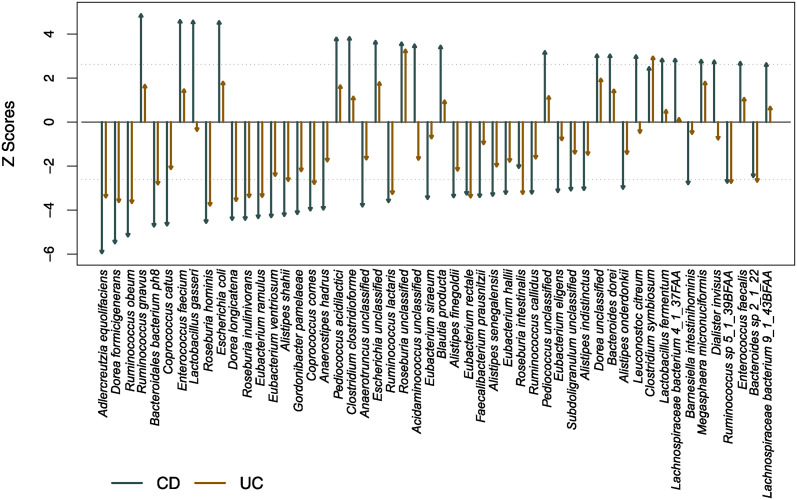
Differentially abundant species in one or more diagnosis in the IBD data analysis at FDR ≤ 0.05. The y-axis indicates z-scores of test results using non-IBD as the reference group. The dotted lines are the Benjamini-Hochberg critical values at FDR = 0.05.

We reanalyzed this dataset using OPTIMEM and a probabilistic index model (PIM), [36] which is the rank equivalent of the general linear model. OPTIMEM identified a subset of non-DA species comprising 43 species, where we used *R* = 2000 and *η* = 0.005. Using this set as a reference, we transformed the proportion of each species into a ratio and implemented a PIM for each species, with diagnosis as a factor of interest and the same covariates used by Franzosa *et al*. [25] We identified 52 DA species in one or more diagnosis. Among them, 44 species coincide with those identified by Franzosa *et al*. [25] This high coincidence was predictable as 45 species have less than or equal to 2 non-zero values in one or two of the groups. DA species identified only by the proposed approach (i.e., OPTIMEM + PIM) were *Acidaminococcus unclassified*, *Bacteroides dorei*, *Leuconostoc citreum*, *Lactobacillus fermentum*, *Megasphaera micronuciformis*, *Dialister invisus*, *Enterococcus faecalis*, *Bacteroides sp 2_1_22*, whereas those identified only by Franzosa *et al*. [25] were *Bifidobacterium breve*, *Alistipes putredinis*, *Holdemania filiformis*, *Lachnospiraceae bacterium 5_1_63FAA*, *Oscillibacter unclassified*, *Lachnospiraceae bacterium 7_1_58FAA*. The directions and magnitudes of identified 52 DA species with respect to non-IBD shown in Fig 6 indicate some compositional effects in the IBD dataset, implying the linear model used by Franzosa *et al*. [25], which does not account for a compositional effect, likely failed to control FDR, as demonstrated in simulation studies.

DA species identified only by the proposed approach were of overall higher abundance than those only identified in Franzosa *et al*. study [25]. All but one were increased in CD but not significantly different in UC subjects, with *Bacteroides sp 2_1_22* decreased in UC with a trend toward decreased abundance in CD. Notably, *D. invisus* was within the top 10 species in overall abundance in this cohort and has been previously shown to have increased levels in subjects with IBD in a separate cohort. [37] Several studies have also correlated increased *E. faecalis* levels to IBD. [38, 39] Only 6 of the top 10 DA taxa identified were the same between both methods. Interestingly, all the top 10 taxa identified in Franzosa *et al*. [25] were negatively associated with IBD, whereas the four non-overlapping taxa identified by the proposed approach were positively associated with IBD (*Ruminococcus gnavus*, *Escherichia coli*, *E. faecium* and *L. gasseri*). These 4 species were highly abundant and were increased in CD patients with no significant difference in UC. Half the top 10 taxa identified by the proposed approach were only significantly different in CD but not UC, whereas 9 of the top 10 taxa identified by Franzosa *et al*. [25] differed in both UC and CD.

#### Human Immunodeficiency Virus (HIV)

Human immunodeficiency virus (HIV) infection is associated with a chronic increase in systemic inflammation and increased intestinal permeability, suggesting that alterations in the gut microbiome may impact this chronic inflammatory state. Chronic inflammation in turn contributes to earlier onset of certain non-AIDS related diseases, such as cardiovascular disease, in people living with HIV (PLH). Several studies have suggested that there are differences in the gut microbiome in PLH compared to healthy controls, but these differences may have been confounded by different sexual practices between comparator groups. To investigate this further, we looked at a well-characterized cross-sectional Ugandan cohort of 39 PLH well-controlled on long-term antiretroviral therapy (ART), 34 ART-naïve PLH, and 37 location-matched HIV negative controls. [26] While differences in bacterial richness and phylogenetic diversity were found when analyzing by immune status, no significant differences were found between PLH on ART, ART-naïve or HIV-negative subjects by Monaco *et al*. [26] OPTIMEM confirmed that there were no DA taxa when comparing these three groups.

#### Microbial communities in the upper respiratory tract

Smoking has been shown to be associated with an increased risk of acute respiratory tract infections that may perturb the upper respiratory tract (URT) microbial communities. To investigate the URT microbial communities, Charlson *et al*. collected microbiome samples from the right and left nasopharynx and oropharynx of 29 smoking and 33 nonsmoking healthy asymptomatic adults. Using 71 taxa with an abundance of >0.2% in at least one airway site, they identified 23 DA taxa between smokers and non-smokers at the nasopharyngeal and/or oropharyngeal microbial samples. [27] They also reported 55 DA taxa between the nasopharyngeal and the oropharyngeal microbial samples, which shows that the majority non-DA taxa assumption can be violated even in two-group comparison. By applying OPTIMEM to these four groups (smokers:oropharynx, smokers:nasopharynx, non-smokers:oropharynx, non-smokers:nasopharynx), we found that all MSS were greater than the upper limit of *MSS*_0_, indicating that all taxa could be DA. In further in-depth analysis, we found this result was purely due to very distinct microbial compositions between the nasopharyngeal and the oropharyngeal microbial samples. OPTIMEM found, in two-group comparisons, no DA taxa between smokers and non-smokers and that all taxa were DA between nasopharynx and oropharynx. The latter is understandable as many taxa only appear either in the nasopharyngeal or oropharyngeal samples, as shown in S7 Fig.

## Discussion

In this manuscript, we establish the minimal assumption required to extract biologically relevant information from compositional data, which is less stringent than existing assumptions, thus being applicable to multigroup and/or longitudinal outcomes. We propose a novel normalization method (OPTIMEM) for high-sparse, high-dimensional compositional data under this minimal assumption. We demonstrated its robustness in identifying a subset of non-DA taxa, which is critical in real world data analysis as we never know the true structure of a given dataset. We showed its beneficial effects on downstream analysis, specifically differential abundance analysis. In addition to these merits, when noise is relatively low, OPTIMEM may provide an empirical way to quantify the validity of its results, i.e., the possibility of DA taxa being included in a determined subset of non-DA taxa.

To demonstrate the beneficial effects of OPTIMEM on downstream analysis, we used differential abundance analysis because the effects of normalization are easily and well manifested in it. As a differential abundance analysis method, we chose rank-based methods (e.g., WR and PIM) because ratios are not well approximated by a normal distribution although log-ratios are. However, after properly treating excessive zeros, which is beyond the scope of this paper, OPTIMEM can be used with any differential abundance analysis methods. It can also be used in other downstream analyses that could be affected by the compositional effect, such as correlation or network analysis. Note that a subset of non-DA taxa determined by OPTIMEM is not necessarily removed in downstream analysis, as their abundance can be treated just as a reference, like the geometric mean in the CLR transformation. Also, note that OPTIMEM does not make any inference. It just utilizes MSS to determine a subset of non-DA taxa, so repeated measures in longitudinal data can be treated as multigroups.

As a real world data application, we first applied OPTIMEM to a cohort of subjects with IBD and healthy controls to determine a subset of non-DA taxa and then used PIM to identify biologically, not compositionally, relevant DA taxa. The proposed approach revealed differences in identified DA species from the initially published analysis. For instance, the proposed approach identified two enterococcal species, *E. faecalis* and *E. faecium* as higher importance than the initial study. Interestingly, several prior studies have shown increased abundance of *Enterococcus* species in subjects with IBD. [40–42] *E. faecalis* levels have been directly correlated with increased disease severity in IBD, Crohn’s disease activity index score, and fecal calprotectin levels, a marker of intestinal inflammation. [39] *E. faecalis* has also been shown to induce IBD and intestinal dysplasia in genetically susceptible mice. [38] *E. faecium* was ranked in the top 10 most significant DA taxa by the proposed approach, but ranked much lower in Franzosa *et al*. [25].

This rank of significance has practical implications, not only with potential probiotic therapeutics, but also for FMT and future mechanistic studies. Small clinical trials of FMT for IBD have shown some success in preventing disease flares, [9] but larger clinical trials are needed. More accurate information on pathogenic or beneficial bacteria in IBD could be used to screen donor stool prior to FMT and increase the success rate of FMT for IBD. This could also allow improved development of probiotic products targeting key taxa beneficial in IBD. Among the DA species only identified by the proposed approach as increased in CD subjects are two that are commercially available as probiotic supplements, *Leuconostoc citreum* and *Lactobacillus fermentum*. [43–45] As several clinical trials of probiotic supplements are currently underway in IBD patients, though not with these organisms, identification of taxa that may exacerbate disease is vital prior to clinical trial of therapeutics, as misidentification could result in worsened disease and increased morbidity if administered. Biologically relevant DA taxa identification could also improve mechanistic studies to ascertain how the pathobionts and beneficial commensals exert their effect on IBD, thus opening new opportunities for directed therapeutics.

Using the proposed approach (i.e., OPTIMEM + PIM), we validated results in the study of PLH showing no DA taxa when comparing PLH on ART, ART-naïve and HIV-negative controls [26]. We also re-examined a cohort of upper respiratory samples from smokers and non-smokers [27] and showed some differences from the original analysis. The proposed approach found only DA taxa between sample collection sites, which could be understandable, but surprisingly no DA taxa by smoking status. These results may be confounded by low sample size at each sampling site or the cross-sectional nature of the sampling, and therefore more studies may be needed to tease out if there are differences induced by smoking and how they impact upper respiratory tract disease.

One disadvantage of OPTIMEM is computation time. In simulation studies, it took a few minutes for a single run on MacBook Pro with 2 GHz Quad-Core Intel Core i5 and 16 GB RAM. It took about 30 minutes for the IBD data analysis, where one taxon was removed at each step. This computation time is controlled by three parameters: the proportion of taxa to be removed *η*, the number of random selection *B*, and the number of random amalgamation *R*. When the number of taxa *p* is less than 1000, removing one taxon at each removal sequence (i.e., *η* < *p*^−1^) is computationally more efficient, as it constrains *B* to the number of remaining taxa. When *p* > 1000, 0.005*p* ≤ *η* ≤ 0.01*p* was observed to balance accuracy and computational time. In this case, *B* should be greater than 2*p*. Unlike *η* and *B*, a larger *R* does not increase computation time substantially, as it often makes OPTIMEM reach the stopping criterion faster. However, gains from increasing in *R* become insignificant when *R* is too large (e.g., *R* > 5*p*). Alternatively, OPTIMEM can be run twice: (1) run with the full *p* taxa at *η* = 0.01*p*, *B* = *p*, and *R* = 2*p*; (2) run with the selected taxa *p*′ in (1) at *η* < *p*′^−1^ and *R* = max(2*p*′, 1000).

## Supporting information

S1 Text Proof of Theorem 1. Asymptotic property of the proposed method.
(PDF)

S2 Text Simulation settings. The parameter settings for the models used in the simulation studies.
(PDF)

S1 Fig Identification of a subset of non-DA taxa (LN model). Scaled densities of selected non-DA taxa for the two approaches (using or not using group membership) with respect to the fold change in abundance of a taxon between two groups, based on LN models. *Y* indicates using group membership and *N* indicates not using group membership.
(TIF)

S2 Fig Favorable setting for existing methods using NB model in balance case. The sample size was 100, and the number of taxa was 100 with 5 to 25 DA taxa randomly selected. The results are based on 100 repetitions. The dotted line indicates FDR = 0.05.
(TIF)

S3 Fig Favorable setting for existing methods using LN model in balance case. The sample size was 100, and the number of taxa was 100 with 5 to 25 DA taxa randomly selected. The results are based on 100 repetitions. The dotted line indicates FDR = 0.05.
(TIF)

S4 Fig Favorable setting for existing methods using LN model in unbalance case. The sample size was 100, and the number of taxa was 100 with 5 to 25 DA taxa randomly selected. The results are based on 100 repetitions. The dotted line indicates FDR = 0.05.
(TIF)

S5 Fig Favorable setting for existing methods using NB model in unbalance case. The sample size was 100, and the number of taxa was 100 with 5 to 25 DA taxa randomly selected. The results are based on 100 repetitions. The dotted line indicates FDR = 0.05.
(TIF)

S6 Fig Favorable setting for existing methods for tertiary outcomes. The sample size was 50 per group, and the number of taxa was 100 with randomly selected number of DA taxa. NB models were used to simulate taxonomic profiles with the majority non-DA constraint. The result is based on 100 repetitions.
(TIF)

S7 Fig Microbial communities in the upper respiratory tract. Mean proportions of taxa in nasopharyngeal vs oropharyngeal microbial samples.
(TIF)
