# Supplementary material for: An optimal normalization method for high sparse compositional microbiome data
Source: PLoS Comput Biol. 2024 Aug 5;20(8):e1012338. doi: 10.1371/journal.pcbi.1012338 (PMC11326560; doi:10.1371/journal.pcbi.1012338)
Supplement: S1 Text — Asymptotic property of the proposed method. (PDF) [file pcbi.1012338.s001.pdf]

An Optimal Normalization Method for High Sparse Compositional Microbiome Data

By Michael B. Sohn, Cynthia Monaco, and Steven R. Gill

Proof of Theorem 1. Recall that $x_g^{(k,b,r)}$ is the mean of log-ratios for group g over n_g at removal sequence (k,b) and random amalgamation step r. Thus, the distribution of $x_g^{(k,b,r)}$ is, by the central limit theorem, well approximated by a univariate normal distribution with sufficiently large n_g, where n_g is the number of samples for group g. Assuming $x_g^{(k,b,r)}$ follows a normal distribution with mean $\mu_g^{(k,b,r)}$ and variance $\sigma^{2(k)}/n_g$ for all g,k,b, and $r=1,\ldots,R$, we have

$$x_i^{(k,b,r)} - x_j^{(k,b,r)} \sim N\left(\mu_i^{(k,b,r)} - \mu_j^{(k,b,r)}, \frac{\sigma^{2(k)}}{n_i} + \frac{\sigma^{2(k)}}{n_j}\right),$$

where $i,j \in \{1,2,\ldots,G\}$. Without loss of generality, we assume $n_1 = n_2 = \cdots = n_g$, and denote

$$V_{ij}^{(k,b,r)} \equiv x_i^{(k,b,r)} - x_j^{(k,b,r)}.$$

Then,

$$W_{ij}^{(k,b,r)} \equiv \frac{\sqrt{n}}{\sqrt{2\sigma^{2(k)}}} V_{ij}^{(k,b,r)} \sim N\big(\delta_{ij}^{(k,b,r)}, 1\big),$$

where

$$\delta_{ij}^{(k,b,r)} = \frac{\sqrt{n}(\mu_i^{(k,b,r)} - \mu_j^{(k,b,r)})}{\sqrt{2\sigma^{2(k)}}}.$$

Hence, $W_{ij}^{2(k,b,r)}$ follows a noncentral chi-squared distribution with one degree of freedom and the noncentrality $\delta_{ij}^{2(k,b,r)}$, i.e.,

$$W_{ij}^{2(k,b,r)} \sim \chi_1^2\big(\delta_{ij}^{2(k,b,r)}\big).$$

Since components are randomly amalgamated to form a log-ratio, $W_{ij}^{(k,b,r)}$ and $W_{ij}^{(k,b,\ell)}$ are independent for all $r,\ell \in \{1,2,\ldots,R\}$. Therefore,

$$W^{2(k,b)} \equiv \sum_{r=1}^{R}\sum_{i=1}^{G-1}\sum_{j=i+1}^{G} W_{ij}^{2(k,b,r)} \sim \chi^2_{RG(G-1)/2}(\lambda^{(k,b)}),$$

where $\lambda^{(k,b)} = \sum_{r=1}^{R}\sum_{i=1}^{G-1}\sum_{j=i+1}^{G}\delta_{ij}^{2(k,b,r)}$. Notice that the noncentrality parameter $\lambda^{(k,b)}$ depends only on the sum of $\delta_{ij}^{2(k,b,r)}$ over $r=1,\ldots,R$; $i=1,\ldots,G-1$; $j=i+1,\ldots,G$, not individual $\delta_{ij}^{2(k,b,r)}$. Thus, we can assume $\delta_{12}^{2(k,b,1)} = \lambda^{(k,b)}$ and $\delta_{ij}^{2(k,b,r)} = 0$ for all other i,j,r and can express

$$W^{2(k,b)} = (Z_1 + \sqrt{\lambda^{(k,b)}})^2 + \sum_{\ell=2}^{RG(G-1)/2} Z_\ell^2 = \sum_{\ell=1}^{RG(G-1)/2} Z_\ell^2 + 2\sqrt{\lambda^{(k,b)}}Z_1 + \lambda^{(k,b)},$$

where $Z_\ell \sim N(0,1)$ for all ℓ. The first term in the second equality is just the sum of $RG(G-1)/2$ independent distributed chi-square variables, each with 1 degree of freedom. Since their means and variances are finite, Lindeberg's condition is satisfied. Hence, by the central limit theorem,

the distribution of the first term converges to a normal distribution with mean $RG(G-1)/2$ and variance $RG(G-1)$ with sufficiently large R. The term $2\sqrt{\lambda}Z_1 + \lambda^{(k,b)}$ follows a normal distribution with mean $\lambda^{(k,b)}$ and variance $4\lambda^{(k,b)}$. Therefore, $W^{2(k,b)}$ converges to a normal distribution with mean $RG(G-1)/2 + \lambda^{(k,b)}$ and variance $RG(G-1) + 4\lambda^{(k,b)}$ with sufficiently large R, i.e.,

$$W^{2(k,b)} \sim N\big(RG(G-1)/2 + \lambda^{(k,b)}, RG(G-1) + 4\lambda\big).$$

Let $(\hat{k}, \hat{b})$ be the optimal indices for (k, b). Then, at removal sequence $\hat{k}$, i.e., when only one DA taxon left in the remaining taxa, $W^{2(\hat{k},\hat{b})} \sim N\big(RG(G-1)/2, RG(G-1)\big)$, as the last DA taxon is removed at $\hat{b}$. Thus, we have

$$W^{2(\hat{k},b)} - W^{2(\hat{k},\hat{b})} \sim N\big(\lambda^{(\hat{k},b)}, 2RG(G-1) + 4\lambda^{(\hat{k},b)}\big) \quad \text{for any } b,$$

and hence

$$\begin{aligned} P(Q^{(\hat{k},b)} > Q^{(\hat{k},\hat{b})}) &= P\bigg(\frac{nR}{2\sigma^{2(\hat{k})}}Q^{(\hat{k},b)} > \frac{nR}{2\sigma^{2(\hat{k})}}Q^{(\hat{k},\hat{b})}\bigg) = P\Big(W^{2(\hat{k},b)} > W^{2(\hat{k},\hat{b})}\Big) \\ &= P\Big(W^{2(\hat{k},b)} - W^{2(\hat{k},\hat{b})} > 0\Big) = 1 - \Phi\bigg(-\frac{\lambda^{(\hat{k},b)}}{\sqrt{2RG(G-1) + 4\lambda^{(\hat{k},b)}}}\bigg), \end{aligned}$$

where $\Phi(\cdot)$ is the cumulative distribution function of the standard normal distribution. Thus, when $\lambda^{(\hat{k},b)}/\sqrt{2RG(G-1) + 4\lambda(\hat{k},b)}$ is sufficiently large, or $\lambda^{(\hat{k},b)} \geq c\sqrt{R}$ for some constant $c > 0$, we have $P(Q^{(\hat{k},b)} > Q^{(\hat{k},\hat{b})}) \approx 1$.

Note that $\lambda^{(\hat{k},b)} = (nR)/(2\sigma^{2(\hat{k})})\mathbb{E}Q^{(\hat{k},b)} - RG(G-1)/2$ so for some constant $c' > c$, we have the following relation:

$$\lambda^{(\hat{k},b)} \geq c\sqrt{R} \quad \Longleftrightarrow \quad \mathbb{E}Q^{(\hat{k},b)} \geq \frac{\sigma^{2(\hat{k})}}{n}\bigg(\frac{c'}{\sqrt{R}} + G(G-1)\bigg).$$

Hence, we can reduce $\mathbb{E}Q^{(\hat{k},b)}$ to the smallest for a given dataset by increasing R, which we have a full control over.

In this proof, we assume $\sigma_g^{2(k,b,r)} = \sigma^{2(k)}$ for all g, b, r. Removing one taxon would not change the variance of an entire composition substantially although the mean proportions of the composition change. Similarly, the variance of a log-ratio constructed by random amalgamation would not vary much. Therefore, the assumption $\sigma_g^{2(k,b,r)} = \sigma_g^{2(k)}$ is reasonable. A homogeneous variance between groups may not be a reasonable assumption. However, we could just use a pooled variance or a maximum variance in the proof as it applies to both $Q^{(\hat{k},b)}$ and $Q^{(\hat{k},\hat{b})}$. There would be no difference in the proof. Only a different value of R would be required to have the same condition for $\lambda^{(\hat{k},b)}$ or $\mathbb{E}Q^{(\hat{k},b)}$. □
