# Supplementary material for: An optimal normalization method for high sparse compositional microbiome data
Source: PLoS Comput Biol. 2024 Aug 5;20(8):e1012338. doi: 10.1371/journal.pcbi.1012338 (PMC11326560; doi:10.1371/journal.pcbi.1012338)
Supplement: S2 Text — The parameter settings for the models used in the simulation studies. (PDF) [file pcbi.1012338.s002.pdf]

An Optimal Normalization Method for High Sparse Compositional Microbiome Data

By Michael B. Sohn, Cynthia Monaco, and Steven R. Gill

S1 Simulation Settings

We simulated taxonomic profiles using two approaches in two cases (balance and unbalance). The first approach used the negative binomial (NB) model whose probability mass function is given by

$$P(X_j = x) = \frac{\Gamma(x+\gamma)}{\Gamma(\gamma)\Gamma(x+1)} \left(\frac{\gamma}{\gamma+\mu}\right)^{\gamma} \left(\frac{\mu}{\gamma+\mu}\right)^{x}, \tag{S1}$$

thus $E(X_j) = \mu$ and $\text{Var}(X_j) = \mu + \mu^2/\gamma$. This approach has been commonly used to simulate omics data, including microbiome data. However, it ignores the correlation between taxa. To incorporate correlations among taxa, the logistic normal (LN) model was used as the second approach. Specifically, we modeled a taxonomic profile as $M_i = \boldsymbol{m}_0 \oplus \boldsymbol{a}^{g_i} \oplus U_i$, where the perturbation operator for two compositions $\boldsymbol{\eta}$ and $\boldsymbol{\zeta}$ in the $p-1$ dimensional simplex space is defined as

$$\boldsymbol{\eta} \oplus \boldsymbol{\zeta} = \left(\frac{\eta_1\zeta_1}{\sum_{j=1}^{p} \eta_j\zeta_j}, \frac{\eta_2\zeta_2}{\sum_{j=1}^{p} \eta_j\zeta_j}, \cdots, \frac{\eta_p\zeta_p}{\sum_{j=1}^{p} \eta_j\zeta_j} \right)^{\top};$$

the power transformation for a composition $\boldsymbol{\eta}$ by a scalar ν is defined as

$$\boldsymbol{\eta}^{\nu} = \left(\frac{\eta_1^{\nu}}{\sum_{j=1}^{p} \eta_j^{\nu}}, \frac{\eta_2^{\nu}}{\sum_{j=1}^{p} \eta_j^{\nu}}, \cdots, \frac{\eta_p^{\nu}}{\sum_{j=1}^{p} \eta_j^{\nu}} \right)^{\top};$$

$\boldsymbol{m}_0$ is a baseline composition; $\boldsymbol{a}$ is a group effect coefficient; a disturbance variable $U_i \sim \text{LN}(\boldsymbol{\mu}, \sigma^2\mathcal{N})$, where $\mathcal{N} = \text{I}_{p-1} + \mathbf{1}_{p-1}\mathbf{1}_{p-1}^{\top}$, I_{p-1} is the identify matrix of size $p-1$, and $\mathbf{1}_{p-1}$ is a vector of $p-1$ ones.

S1.1 Identification of a Subset of Non-DA Taxa

To assess the performance of OPTIMEM in selecting a subset of non-DA taxa with and without using group membership, we randomly selected a number of DA taxa from 5 to 50 out of 100 taxa and randomly generated their fold changes for two groups $g = 1, 2$. We then simulated a taxonomic profile using LN models with these fold changes. In each run, a sample size of 100 was used for both groups.

S1.2 Favorable Settings for Existing Methods

We randomly generated a number of DA taxa from 5 to 25 out of 100 taxa and simulated a taxonomic profile using an NB or LN model in the balance or unbalance case. The mean counts of non-DA taxa were first randomly selected from $\{1, 2, \ldots, 100\}$. Then, the mean counts of the randomly selected k DA taxa in one group were replaced with randomly selected mean counts for the balance case, i.e.,

$$g_1 = (x_1, x_2, \ldots, x_k, x_{k+1}, \ldots, x_p)$$
$$g_2 = (x'_1, x'_2, \ldots, x'_k, x_{k+1}, \ldots, x_p)$$

For the unbalance case, we multiplied the k mean counts by randomly selected values, i.e.,

$$g_1 = (x_1, x_2, \dots, x_k, x_{k+1}, \dots, x_p)$$
$$g_2 = (x_1 a_1, x_2 a_2, \dots, x_k a_k, x_{k+1}, \dots, x_p),$$

where $a_j \in \{1.5, 1.6, \dots, 3.0\},\ j = 1, \dots, k$.

For the LN model, the baseline composition $\boldsymbol{m}_0$ was generated using the constraining operator with randomly selected counts from $\{1, 2, \dots, 5\}$, and a group effect coefficient $\boldsymbol{a}$ was generated by $\mathscr{C}(\boldsymbol{x}, \mathbf{1}_{p-n_{DA}})$, where $\boldsymbol{x}$ is a vector of k randomly selected group effects from $\{0.2, 0.3, \dots, 0.8, 1.5, 2, \dots, 5\}$ for the balance case and $\{1.5, 1.6, \dots, 3.0\}$ for the unbalance case. Group membership g_i was randomly generated from Bernoulli distribution with parameter 0.5. The covariance matrix of the disturbance variable in the LN model was set at $\mathcal{N}$, i.e., $\sigma^2 = 1$, and the sample size n of 100 was used.

S1.3 Antagonistic Settings for Existing Methods

We simulated taxonomic profiles using NB models in three artificial scenarios, where some or all existing methods perform poorly. The first two scenarios do not violate any assumptions imposed by any existing methods, but the last scenario violates the majority non-DA assumption imposed by most existing methods. In all scenarios, we used a sequencing depth randomly generated from $\{1, 2, \dots, 10\}$ for each sample, dispersion parameter $\gamma = 1$, and the sample size $n = 100$. For the mean counts of settings 1 and 2, we first randomly generated the number η_j of DA taxa having the same mean counts from $\{1, 2, \dots, 6\}$ and then constructed $\boldsymbol{\mu}_1$. The mean counts for $\boldsymbol{\mu}_2$ was constructed by multiplying $\boldsymbol{\mu}_1$ by randomly generated effect sizes $\boldsymbol{\alpha}$.

- Scenario 1: There exist sets of DA taxa subcompositionally equivalent between two groups under the balance case.

  $$\boldsymbol{\mu}_1 = \big(\{\text{rep}(\mu_j, \eta_j); j = 1, \dots, k\},\ \mu_\ell, \dots, \mu_p\big)$$
  $$\boldsymbol{\mu}_2 = \big(\{\text{rep}(\alpha_j \mu_j, \eta_j); j = 1, \dots, k\},\ \mu_\ell, \dots, \mu_p\big),$$

  where rep() is a replicate function, e.g., rep$(a, 3) = (a, a, a)$, μ_j was randomly generated from $\{1, 2, \dots, 10\}$, $\ell = \sum_j \eta_j + 1$, and α_k was generated from a uniform distribution over the interval $[0.25, 3.0]$ with a constraint $\sum_j \mu_{1j} = \sum_j \mu_{2j}$.

- Scenario 2: There exist sets of DA taxa subcompositionally equivalent between two groups under the unbalance case.

  $$\boldsymbol{\mu}_1 = \big(\{\text{rep}(\mu_j, \eta_j); j = 1, \dots, k\},\ \mu_{k+1}, \dots, \mu_p\big)$$
  $$\boldsymbol{\mu}_2 = \big(\{\text{rep}(\alpha_j \mu_j, \eta_j); j = 1, \dots, k\},\ \mu_{k+1}, \dots, \mu_p\big),$$

  where where μ_j was randomly generated from $\{1, 2, \dots, 10\}$ and α_j was generated from $\{2.0, 2.5, \dots, 4.0\}$

- Scenario 3: The majority non-DA taxa assumption is violated under the unbalance case.

S1.4 Three-Group Outcome Settings

We first simulated taxonomic profiles using NB and LN models mixing the balance and unbalance cases. The number of DA-taxa across three groups was randomly selected from $\{10, 11, \dots, 45\}$ out of 100 taxa. The sample size was 50 per group. Note that these settings do not violate any assumptions of existing methods. We then assessed the performance of the proposed method at different fold changes between groups when the number of DA taxa ranged from 10 to 70, i.e., some simulated taxonomic profiles violated the majority non-DA taxa assumption. Specifically, We first

randomly selected mean counts $\boldsymbol{\mu}$ of 100 taxa from $(1, 2, \ldots, 100)$. We then randomly selected a number $n_{sel}^{(g)}$ of DA-taxa for each of three groups and randomly selected their mean counts $\boldsymbol{\mu}^{(g)}$ from $(1, 2, \ldots, 100)$. We replaced the first $n_{sel}^{(g)}$ mean counts in $\boldsymbol{\mu}$ with $\boldsymbol{\mu}^{(g)}$. Using these mean counts for each group and dispersion parameter $\gamma = 1$, we simulated taxonomic profiles using NB models. We used the sample size of 50 for each group.
