# Supplementary figures and images for: An optimal normalization method for high sparse compositional microbiome data
Source: PLoS Comput Biol. 2024 Aug 5;20(8):e1012338. doi: 10.1371/journal.pcbi.1012338 (PMC11326560; doi:10.1371/journal.pcbi.1012338)

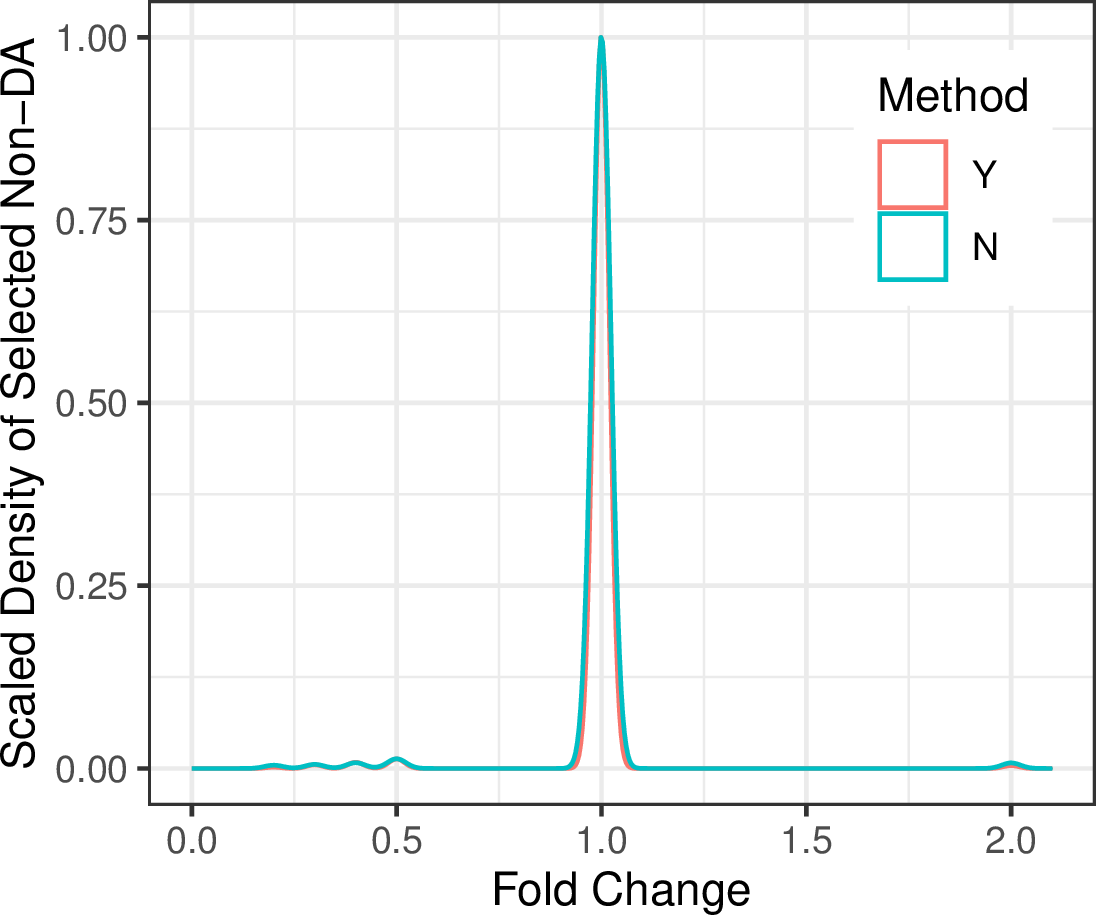

Supplement: S1 Fig — Scaled densities of selected non-DA taxa for the two approaches (using or not using group membership) with respect to the fold change in abundance of a taxon between two groups, based on LN models. Y indicates using group membership and N indicates not using group membership. (TIF) [file pcbi.1012338.s003.tif]

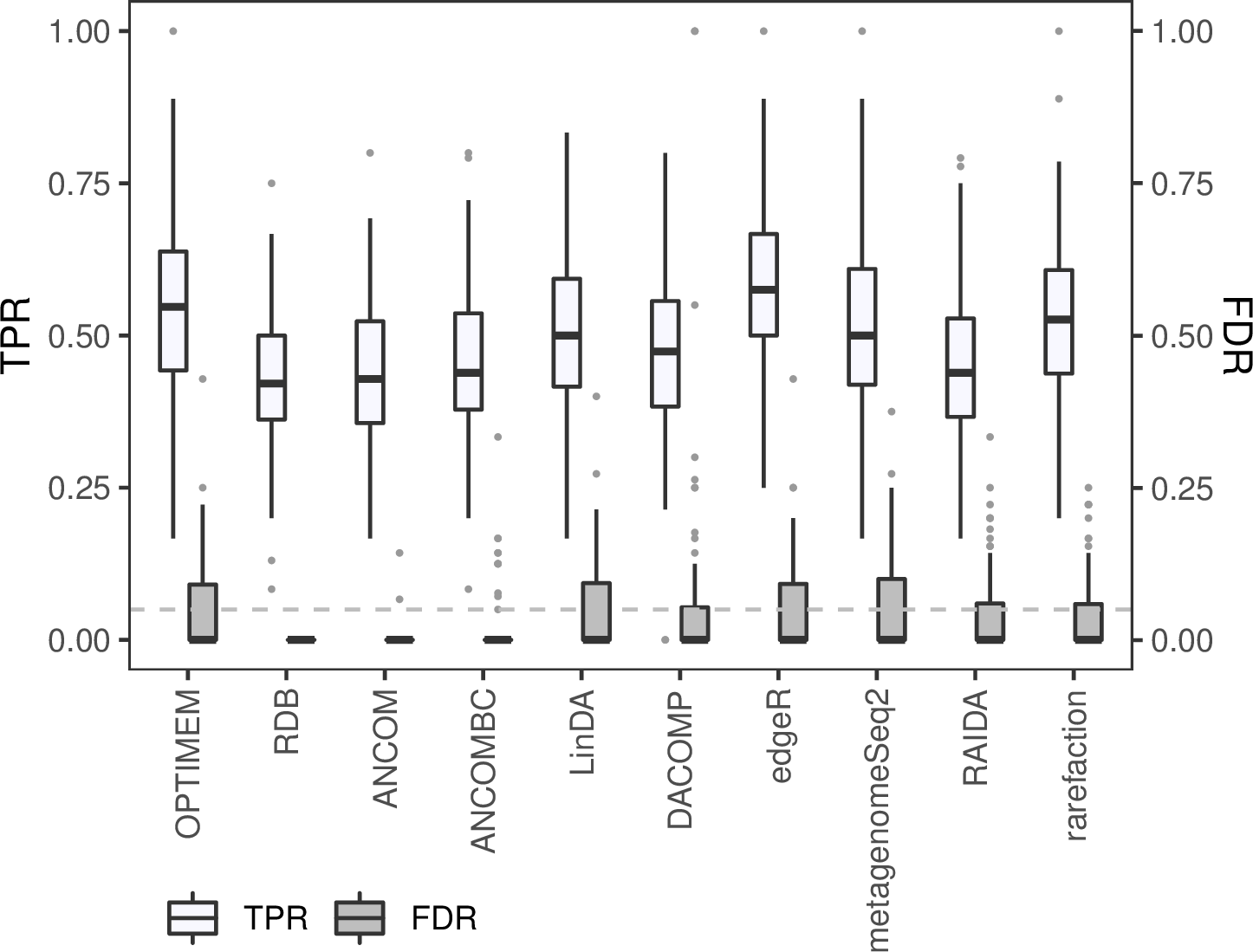

Supplement: S2 Fig — The sample size was 100, and the number of taxa was 100 with 5 to 25 DA taxa randomly selected. The results are based on 100 repetitions. The dotted line indicates FDR = 0.05. (TIF) [file pcbi.1012338.s004.tif]

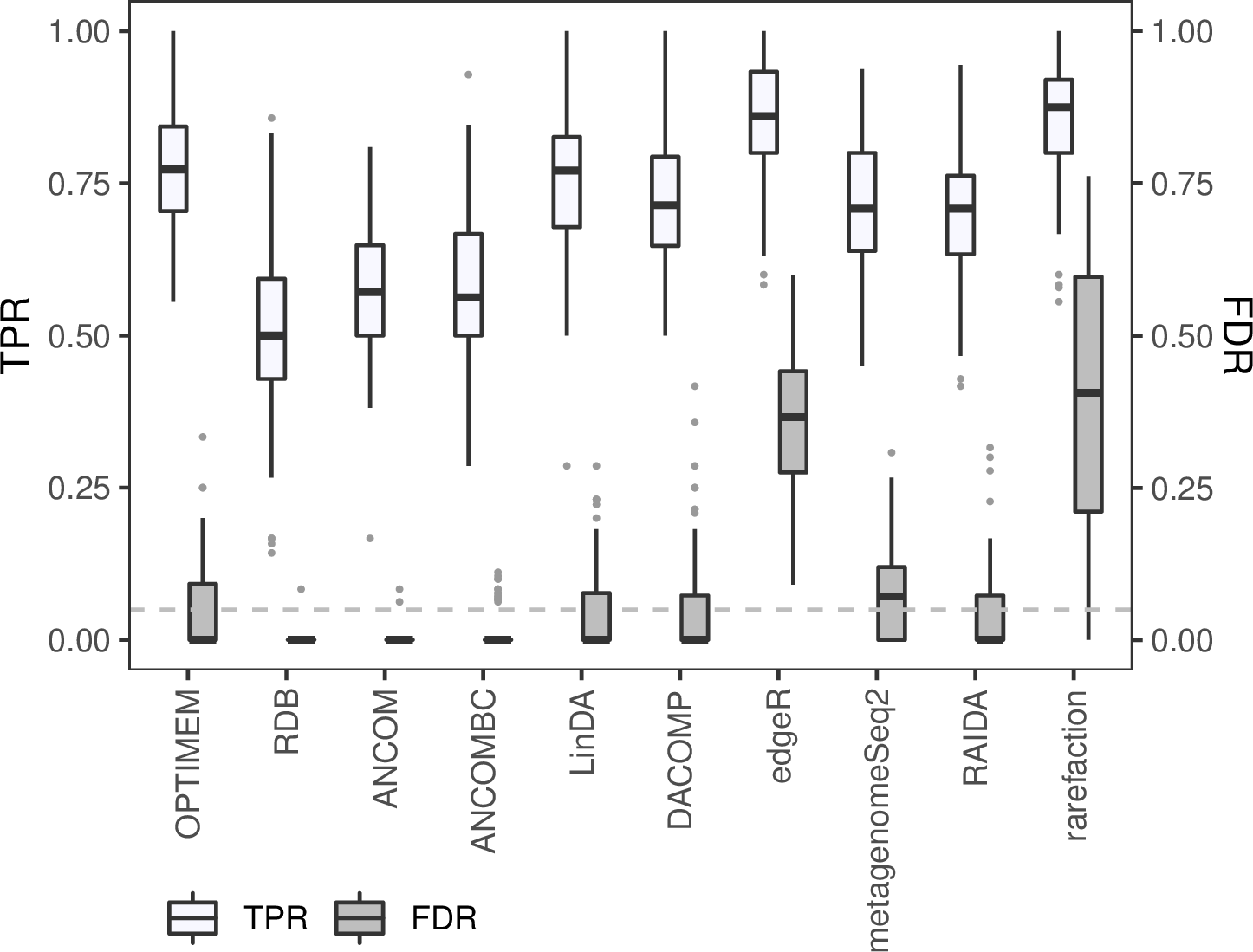

Supplement: S3 Fig — The sample size was 100, and the number of taxa was 100 with 5 to 25 DA taxa randomly selected. The results are based on 100 repetitions. The dotted line indicates FDR = 0.05. (TIF) [file pcbi.1012338.s005.tif]

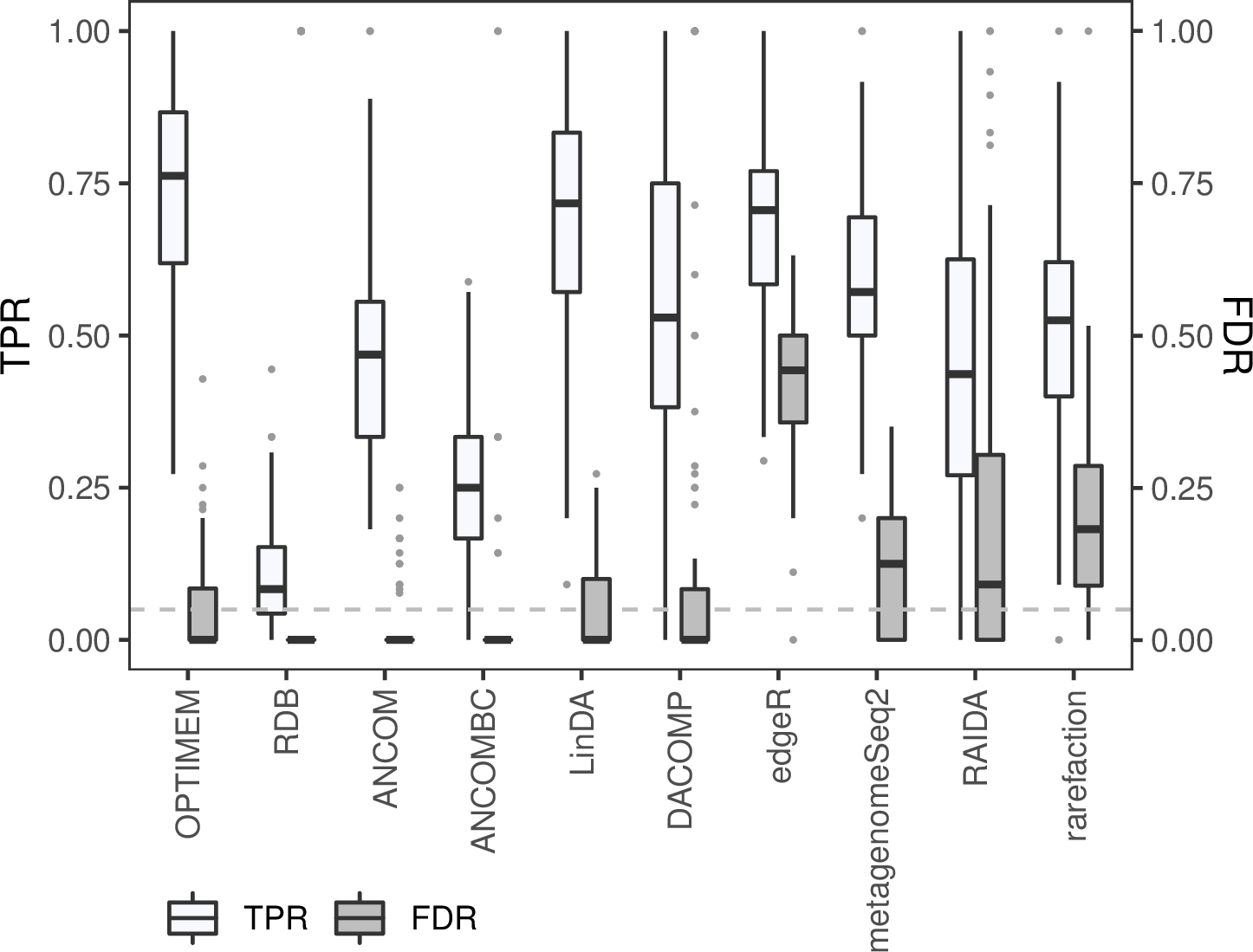

Supplement: S4 Fig — The sample size was 100, and the number of taxa was 100 with 5 to 25 DA taxa randomly selected. The results are based on 100 repetitions. The dotted line indicates FDR = 0.05. (TIF) [file pcbi.1012338.s006.tif]

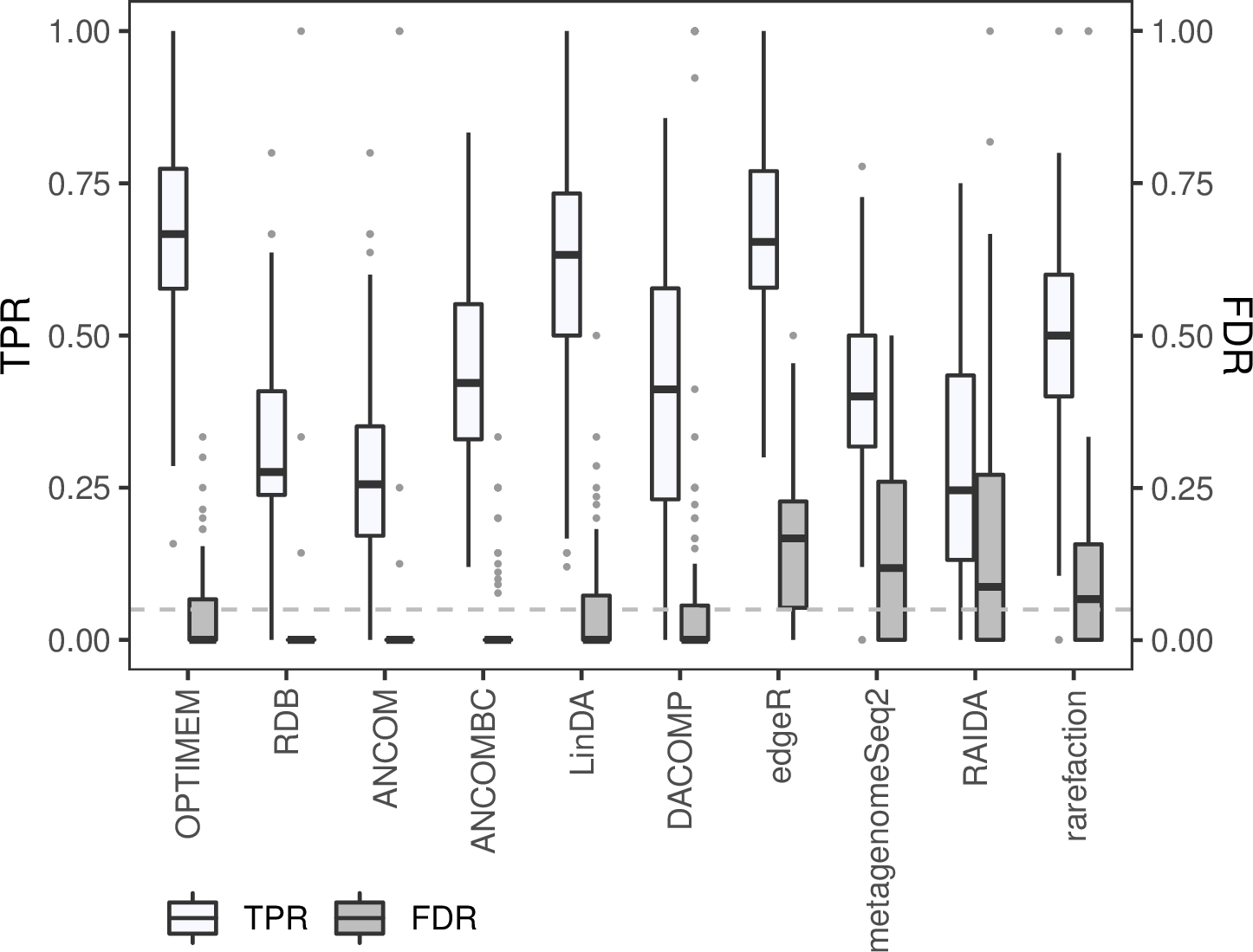

Supplement: S5 Fig — The sample size was 100, and the number of taxa was 100 with 5 to 25 DA taxa randomly selected. The results are based on 100 repetitions. The dotted line indicates FDR = 0.05. (TIF) [file pcbi.1012338.s007.tif]

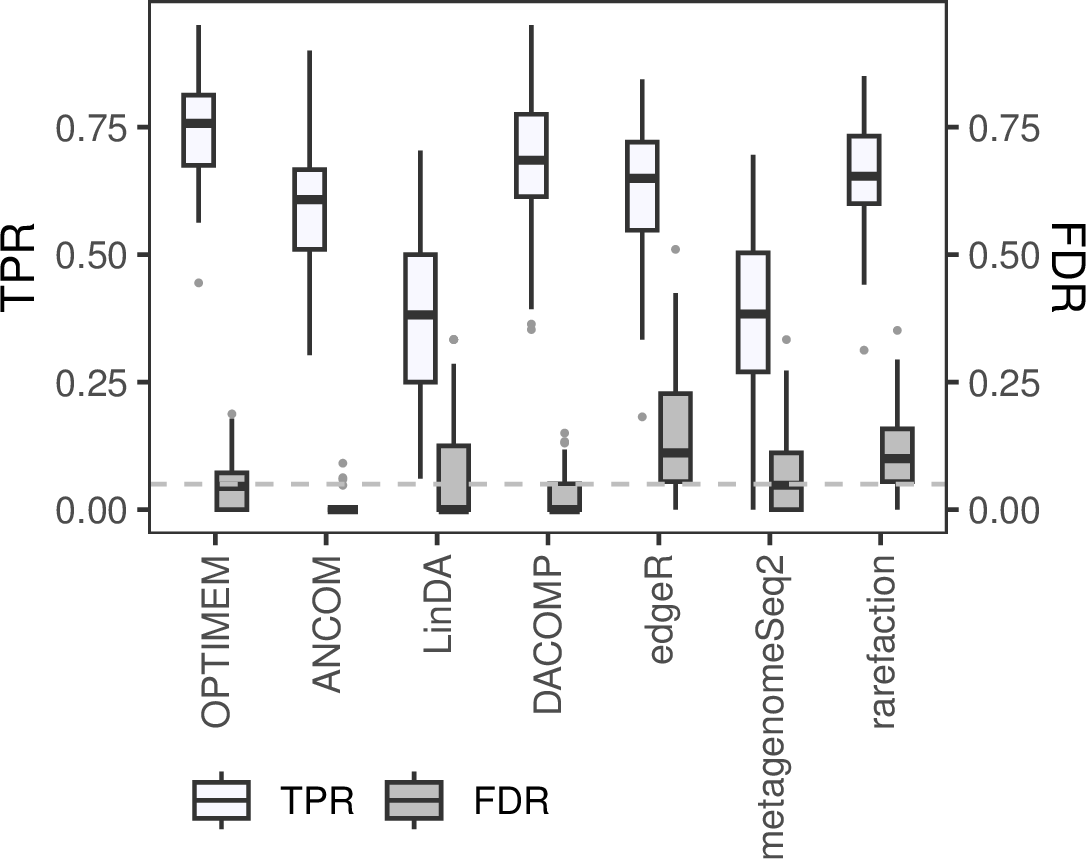

Supplement: S6 Fig — The sample size was 50 per group, and the number of taxa was 100 with randomly selected number of DA taxa. NB models were used to simulate taxonomic profiles with the majority non-DA constraint. The result is based on 100 repetitions. (TIF) [file pcbi.1012338.s008.tif]

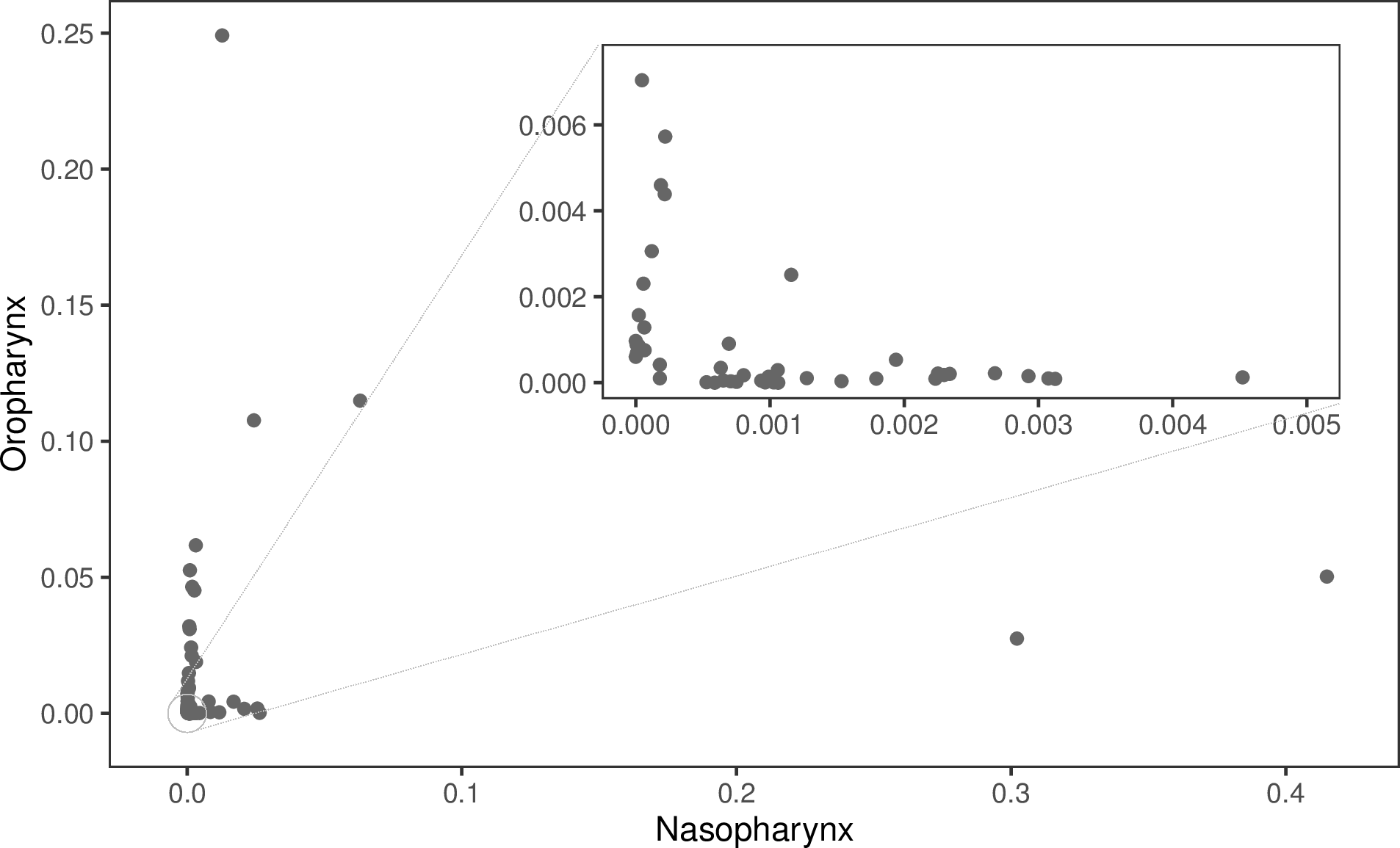

Supplement: S7 Fig — Mean proportions of taxa in nasopharyngeal vs oropharyngeal microbial samples. (TIF) [file pcbi.1012338.s009.tif]
